# Distribution characteristics of salivary cortisol measurements in a healthy young male population

**DOI:** 10.1186/s40101-015-0068-0

**Published:** 2015-08-19

**Authors:** Hiromitsu Kobayashi, Yoshifumi Miyazaki

**Affiliations:** Center for Environment, Health and Field Sciences, Chiba University, Chiba, Japan

**Keywords:** salivary cortisol, distribution, skewness, kurtosis, floor-effect

## Abstract

**Background:**

Salivary cortisol has been used in various fields of science as a non-invasive biomarker of stress levels. This study offers the normative reference values of cortisol measurement for healthy young males.

**Findings:**

Salivary cortisol levels were measured in 267 healthy young males (age: 21.7 ± 1.5 years) in the early morning on two consecutive days and were analyzed by radioimmunoassay. Frequency distribution analysis was conducted with mean values of the measurements taken on the 2 days. The mean salivary cortisol level was 20.39 ± 7.74 nmol/l (median: 19.31 nmol/l). The skewness and kurtosis of the distribution of the raw data were 0.72 and 0.68, respectively. They were both improved by a square root transformation but not by a logarithmic transformation.

**Conclusions:**

The skewness of the distribution for salivary cortisol measured in the early morning is considerably smaller than that previously reported from afternoon measurements. A “floor effect” may be an explanation for the difference in the distribution characteristics of salivary cortisol.

## Background

Cortisol is a steroid hormone produced by the zona fasciculata of the adrenal cortex to regulate carbohydrate, fat, and protein metabolism. The cortisol level is considered to be an indicator of the activity of the hypothalamic-pituitary-adrenal (HPA) axis, which is responsible for corticotropin-releasing hormone (CRH) and adrenocorticotropin (ACTH) secretions. The HPA system is among the major stress response systems of the human biology. The ACTH-mediated cortisol secretion induces an increase in blood pressure and glucose levels and immune system suppression. These physiological reactions are called “fight or flight” response. Thus, cortisol measurements have been used as a biological indicator of stress.

Both acute (e.g., skydiving [1]) and chronic (e.g., work overload [2]) stress can lead to elevations in blood levels of cortisol. Cortisol additionally increases due to chronic diseases, e.g., type II diabetes [3], Cushing’s syndrome [4], or psychiatric disorders [5].

Chronic exposure to elevated cortisol may cause some pathogenic processes [6]. This cumulative effect of stress responses on physiological functions is called allostatic load [7]. Thus, cortisol is an effective allostasis/allostatic load indicator [6].

Cortisol level analysis was performed with blood serum. The disadvantage of this method is not only costly but it may also lead to falsely elevated cortisol levels because blood collection alone may activate the HPA axis [8]. On the other hand, cortisol is also detected in hair, urine and saliva. Hair cortisol concentration provides an advantage for evaluating chronic stress because hair growth is slow. Conversely, salivary cortisol levels indicate acute response to stress and serum cortisol [9]. Salivary cortisol concentration was directly proportional to the serum unbound cortisol concentration [10]. The recent collection and analytical protocol development for human salivary cortisol expanded the application range of the measurements. For instance, salivary cortisol is used as a neuroendocrine marker of stress in various fields of science, including physiological anthropology [11, 12].

Several studies documented salivary cortisol in large populations of healthy individuals [13], including 6470 healthy adults aged 45 years [14] and 1768 children [15]. Thus, most large-scale studies investigated middle-aged adults (>40 years) or pre-adolescent children (10–13 years). However, none of them determined normal cortisol levels in the saliva of young adults (20–30 years). Therefore, the present study determined the normative salivary cortisol values in 267 healthy Japanese males, with special reference to the characteristics of the concentration distribution.

## Methods

### Participants

The 267 Japanese male students who participated in the present study constitute a subset of the participants enrolled in our previous study on 417 young Japanese male students [16]. None reported physical or psychiatric disorder history. All participants were non-smokers, and alcohol or dietary supplement intake on the day before the measurement was forbidden. The study was conducted under the regulations of the Institutional Ethical Committee of the Forestry and Forest Products Research Institute and the Center for Environment, Health and Field Sciences, Chiba University, Japan.

### Saliva collection and analysis

Saliva samples were collected from each participant using a Salivette (No. 51.1534; Sarstedt, Numbrecht, Germany). The saliva was collected before breakfast, approximately 20–40 min after awakening (6:30–7:30 a.m.) and before they brushed their teeth. Each participant rested for 1 min in a sitting position before saliva collection. The measurement was repeated the following day. The samples were immediately frozen and transported to the laboratories of SRL Inc. (Tokyo, Japan) for cortisol concentration measurement. An aliquot of 0.25 ml of saliva was analyzed by radioimmunoassay.

### Statistical analysis

The initial cortisol level (COR-raw) measurements were converted using natural logarithmic (COR-log) and square root (COR-sqrt) scales. The mean value of the measurements taken on two consecutive days from each participant was used to characterize the distribution. The mean, median, standard deviation (SD), coefficient of variation (CV; SD/mean), skewness (symmetry parameter), kurtosis (peakedness parameter), and 95 % confidence interval (95 % CI) were calculated for the raw data and numerically transformed cortisol levels.

## Results

The participants were healthy young adults (mean, 21.8 ± 1.5 years; range, 20–29) with normal and consistent height (mean, 171.9 ± 5.4 cm; 155–188 cm) and body weight (mean, 64.4 ± 9.6 kg; range, 47–110 kg) (Table 1).

**Table 1:** Demographic parameters of the participants

	Age (years)	Height (cm)	Weight (kg)
Mean	21.8	171.9	64.4
SD	1.5	5.4	9.6
Max	29	188	110
Min	20	155	47

*SD* standard deviation

The quantitative analysis of the raw (COR-raw) and numerically transformed (COR-log and COR-sqrt) salivary cortisol levels are summarized in Table 2. Although the COR-raw exhibited 38 % of CV, the numerical transformations reduced the CVs to 14 % (COR-log) and 20 % (COR-sqrt).

**Table 2:** Characteristics of the distribution of the raw and numerically transformed salivary cortisol levels in healthy young male adults

	COR-raw (nmol/l)	COR-log ln (nmol/l)	COR-sqrt (nmol/l)
Mean	20.39	2.92	4.41
Median	19.31	2.94	4.37
SD	7.74	0.41	0.86
CV	38 %	14 %	20 %
95 % CI	7.59–39.59	1.99–3.67	2.72–6.28
Skewness	0.73	−0.63	0.12
Kurtosis	0.68	1.14	0.20

The skewness and kurtosis of a normal distribution are both zero

*SD* standard deviation, *CV* coefficient of variation (SD/mean), *CI* confidence interval, *skewness* parameter of symmetry, *kurtosis* parameter of peaked (positive) or flat (negative) distribution

**Fig. 1:**
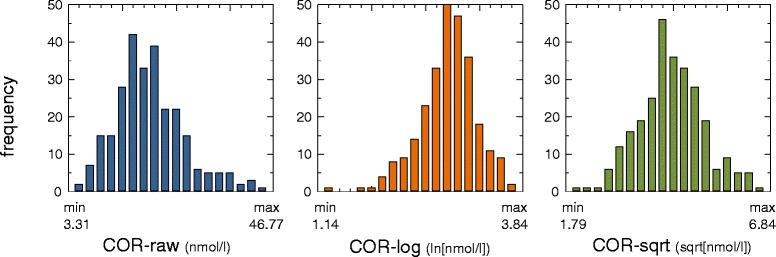
Frequency distributions of the raw and numerically transformed salivary cortisol levels of healthy young male adults. The raw data (*COR-raw*) exhibit a right-skewed distribution, whereas the logarithmic data (*COR-log*) exhibit a left-skewed distribution. In contrast, the square root transformation (*COR-sqrt*) generated a nearly symmetrical distribution

The frequency distribution histograms were compared to test the data transformation impact on the inter-individual variability in cortisol levels (Fig. 1). The COR-raw histogram exhibited a slightly right-skewed and peaked curve, consistent with the skewness of 0.73 and the kurtosis of 0.68. In contrast, the COR-log histogram was left-skewed because of a skewness of −0.63 and presented a kurtosis of 1.14. Interestingly, the square root conversion generated a nearly normal distribution because the COR-sqrt histogram only exhibited skewness and kurtosis values of 0.12 and 0.20, respectively.

## Discussion

Cortisol secretion follows a circadian rhythm, whereby circulating levels are low or undetectable at midnight, accumulate overnight to peak 30–45 min after awakening, and then decline slowly throughout the day [8]. This peak phenomenon is called cortisol awakening response (CAR). Thus, samples collected in the early morning provide the most sensitive measurements of cortisol levels. The present study shows that saliva samples collected from healthy young Japanese adults in the early morning contain a mean cortisol level of 20.39 nmol/l. These results are mostly consistent with the mean salivary cortisol level measured in the early morning in 45-year-old men in England (21.01 nmol/l) [14] and in elderly subjects in Rotterdam (*<*65 years; 18.4 nmol/l) [17]. Furthermore, Hansen et al. [18] reported a 95 % CI for salivary cortisol level of 7.6–39.4 nmol/l in the early morning for adults in Denmark, which is remarkably similar to the 95 % CI calculated in the present study (7.59–39.59). Collectively, these results demonstrate that salivary cortisol level measurements in the early morning are highly consistent, even between adult populations of distinct ethnicities.

Mean height and body mass of the participants were almost identical with the means of Japanese males aged 22 years (171.3 ± 4.7 cm and 65.0 ± 10.7 kg) [19]. Therefore, the sample population of this study can be considered as representatives of physical traits. For socioeconomic status, the sample population may not be a good representative of the Japanese population because all participants were college students. This is a limitation in interpreting the present results.

Little is known about the distribution characteristics of salivary cortisol levels in large populations. The present study demonstrates that the skewness of the data distribution for salivary cortisol measured in the early morning is considerably smaller (skewness, 0.73) than for afternoon measurements (skewness, 3.5–4.8) that previously reported [20, 21]. Kiess et al. [22] investigated the diurnal change of salivary cortisol in various age groups. The median of the morning cortisol was located in approximately the middle of the 90 % interval, suggesting an approximate symmetrical distribution. Conversely, evening cortisol median shifted close to the fifth percentile value, suggesting a right-skewed distribution. The median shift toward the lower limit of the variation was consistently observed in adults, adolescents, children, and neonates. Similar results were additionally demonstrated in children (mean age = 10.9 years) by Carrion et al. [23]. Thus, the difference in skewness could be attributed to measurement time of day, rather than differences in age, gender, or ethnicity.

Even if the distribution of peak cortisol in the early morning is almost symmetrical, the lowered cortisol level in the afternoon does not necessarily present a symmetrical distribution. A “floor effect” [24] prohibits negative values in cortisol, resulting in a distorted distribution with a shorter left tail (right skewness). This phenomenon may explain the difference in skewness of the data distributions obtained with afternoon and morning samples.

In conclusion, the present study demonstrated that the skewness of salivary cortisol concentration measured in the early morning is relatively smaller compared with that measured in the afternoon. Although logarithmic transformations have been proposed for salivary cortisol in the previous studies [13, 18, 21], we recommend a square root transformation for morning salivary cortisol measurements.

## Abbreviations

ACTH: adrenocorticotropin
CAR: cortisol awakening response
CI: confidence interval
COR-log: natural logarithmic transformed salivary cortisol
COR-raw: raw salivary cortisol
COR-sqrt: square root transformed salivary cortisol
CRH: corticotropin-releasing hormone
CV: coefficient of variation
HPA: hypothalamic-pituitary-adrenal
SD: standard deviation
